# Two Chinese siblings of combined oxidative phosphorylation deficiency 14 caused by compound heterozygous variants in *FARS2*

**DOI:** 10.1186/s40001-022-00808-7

**Published:** 2022-09-26

**Authors:** Liangshan Li, Jianhua Ma, Jingli Wang, Liping Dong, Shiguo Liu

**Affiliations:** 1 Medical Genetic Department, The Affiliated Hospital of Qingdao University, Qingdao, 266003 China; 2 Department of Reproductive Medicine, The Affiliated Hospital of Qingdao University, Qingdao, China; 3 Neonatal Disease Screening Center, Zibo Maternal and Child Health Hospital, Zibo, 255000 China

**Keywords:** Combined oxidative phosphorylation deficiency 14, *FARS2*, Compound heterozygous variants, Whole exome sequencing

## Abstract

**Background:**

As a rare mitochondrial disease, combined oxidative phosphorylation deficiency 14 (COXPD14) is caused by biallelic variants in the phenylalanyl-tRNA synthetase 2, mitochondrial gene (*FARS2*) with clinical features of developmental delay, an elevated lactate level, early-onset encephalopathy, liver failure, and hypotonia. The objectives of this study were to analyze the clinical and molecular features of two Chinese siblings affected with COXPD14, and to review relevant literature.

**Methods:**

Mutation screening was performed by whole exome sequencing (WES) in combination with Sanger sequencing validation to identify the disease-causing variants of the two patients.

**Results:**

The two siblings presented with severe clinical features and both progressed aggressively and failed to survive after treatment abandonment. We identified two compound heterozygous *FARS2* variants c.925G>A p.Gly309Ser and c.943G>C p.Gly315Arg in this proband, which were inherited from the unaffected father and mother, respectively. In addition, Sanger sequencing confirmed that the elder affected sister carried the same compound heterozygous variants. The c.925G>A p.Gly309Ser variant is known and commonly reported in COXPD14 patients, while c.943G>C p.Gly315Arg is a novel one. Neither of the variants was found in 100 Chinese healthy controls. Both variants were classified as “deleterious” and were located in the highly conserved regions of the protein. The above results suggested that the two variants were likely causative in this COXPD14-affected pedigree.

**Conclusions:**

Our study expands the mutation spectrum of *FARS2* and highlights the importance of genetic testing in the diagnosis of diseases with a wide variety of phenotypes, especially in the differential diagnosis of diseases.

## Introduction

As the consequences of defects in nuclear DNA (nDNA) or mitochondrial DNA (mtDNA), mitochondrial diseases are a highly heterogeneous group of inherited metabolic disorders characterized by a broad phenotypic spectrum, differential disease course, varying age of onset, and diverse consequences[1, 2]. They are due to impairments in mitochondrial respiratory chain oxidative phosphorylation (OXPHOS) function, which in turn have effects on multiple systems of the human body [3, 4]. The estimated prevalence of adult mitochondrial diseases given rise to causative variants of mitochondrial and nuclear genomes is 1 per 4300 individuals [5].

Combined oxidative phosphorylation deficiency (COXPD) is a severe disorder belonging to mitochondrial diseases with an autosomal recessive inheritance pattern. To date, COXPD has been divided into 51 types (COXPD1–COXPD51) based on different disease-causing genes in Online Mendelian Inheritance in Man (OMIM). Among these, combined oxidative phosphorylation deficiency 14 (COXPD14, MIM: 614946) is caused by biallelic variants in phenylalanyl-tRNA synthetase 2, mitochondrial (*FARS2*) (MIM: 611592). The clinical features encompass developmental delay, an elevated lactate level, early-onset epileptic encephalopathy, microcephaly, thin corpus callosum, brain atrophy, liver disease, and axial hypotonia [6, 7].

*FARS2 *is a nuclear gene that maps to chromosome 6p25.1 and spans over 510 kb with seven exons, six of which are coding [6, 8]. The encoded protein mitochondrial phenylalanyl-tRNA synthetase (mtPheRS) is composed of 451 amino acids and could transfer phenylalanine to its cognate mitochondrial tRNA, which is essential for the translation of mitochondrial DNA-encoded proteins [9]. The four major domains of mtPheRS consist of an N-terminal domain (residues 37–83), a catalytic aminoacylation domain (residues 84–325), a linker domain (residues 326–358), and a C-terminal anticodon-binding domain (residues 359–451) [6, 10]. Review of the literature demonstrates that homozygous or compound heterozygous *FARS2* pathogenic variants are responsible for three distinct clinical phenotypes, including early-onset epileptic encephalopathy, spastic paraplegia, and the latest report of juvenile-onset refractory epilepsy [11].

In this study, we described the clinical presentations of two Chinese siblings affected by COXPD14 and found two compound heterozygous variants in *FARS2* by utilizing whole exome sequencing (WES) combined with Sanger sequencing validation, which further expand the molecular and phenotypic spectrum of COXPD14 caused by genetic defects in *FARS2*. Additionally, we retrospectively reviewed and summarized the clinical and molecular data of the reported patients with *FARS2* variants.

## Materials and methods

### Patients

The male proband (Patient II: 2, Fig. 1) and his families were recruited and examined in Zibo Maternal and Child Health Hospital. This study was approved by the Ethics Committee of the Affiliated Hospital of Qingdao University and Zibo Maternal and Child Health Hospital. Peripheral blood samples were collected from the proband, his elder sister and parents, and 100 healthy controls of Chinese Han origin after informed consent was obtained.

**Fig. 1:**
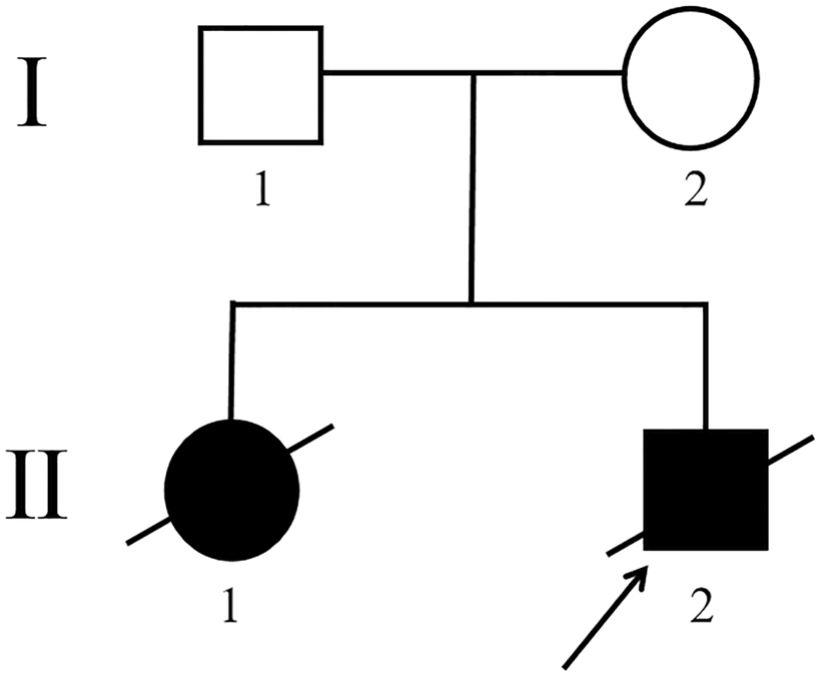
Pedigree of the family with COXPD14. The black arrow denotes the proband

### WES

Following genomic DNA extraction, qualified DNA sample was randomly sheared to generate 180–280 bp DNA fragments, which were selected for the preparation of DNA libraries. The currently identified 6259 genetic phenotypes by OMIM were detected, and a total of 1839 genes associated with clinical phenotypes of the proband were focused. The libraries were hybridized with biotin-labeled probes in liquid phase; then the streptavidin magnetic beads were used to bind with biotin-containing target fragments for the capture of the exons of these genes. The paired-end reads of 150 bp sequencing was performed on an Illumina NextSeq 500 sequencer (Illumina, San Diego, CA, USA) after enrichment and quality inspection of the libraries.

### Sequencing data analysis

Low-quality reads and raw reads with adaptor were removed. The Burrows-Wheeler Aligner (BWA) software was used to align the clean reads to the human reference genome (hg19). Subsequently, the alignment results were sorted using SAMtools and duplicated reads were marked with the Picard software for the statistical analyses of sequencing depth and coverage. On the completion of these, single nucleotide polymorphisms (SNPs) and insertions and deletions (InDels) variation sites were detected and annotated. Filter SNPs and InDels with minimum allele frequency (MAF)>0.02. Pathogenicity assessment of the nonsynonymous variants was performed by *in silico* analysis.

### Sanger sequencing validation

The likely pathogenic variants detected in this proband were confirmed among all available family members and 100 Chinese healthy subjects by Sanger sequencing. Primers involving the mutation sites were designed by Primer Premier version 5.0 software. The primer sequences for PCR amplification were as follows: forward 5'-GAGGGCAGTCCGGAATATGG-3' and reverse 5'-CCTGTCGATCCTTGACAGCC-3'. Sequencing primer sequence was as follows: reverse 5'-CCTGTCGATCCTTGACAGCC-3'. After Polymerase Chain Reaction (PCR) and agarose gel electrophoresis, the gel-recovered PCR products were analyzed on an ABI 3730 analyzer (Applied Biosystem). The sequencing data were aligned to the reference sequence on the National Center Biotechnology Information (NCBI) website for the determination of the mutation sites.

## Results

### Clinical manifestations

The male proband (Patient II: 2, Fig. 1) was born to healthy and nonconsanguineous Chinese parents. No family history of genetic disorders was found in both maternal and paternal families. He was born at a gestational age of 37 weeks by cesarean section with a birth weight of 3500 g and head circumference of 33 cm. He postnatally displayed poor mental response and was initially admitted to the hospital for shortness of breath, foaming at the mouth accompanied by moaning without obvious inducement for 1 h after birth. No improvement was observed after airway clearing stimulation. Physical examination showed clear consciousness, poor mental reaction, less ruddy skin, shortness of breath, dry and moist rales, thick breath sounds in both lungs, no pathologic murmurs in the valve areas, thick skin with many folds, popliteal angle< 90°, hypotonia of the limbs, and diminished neonatal reflexes, such as sucking, swallowing, and hugging.

**Fig. 2:**
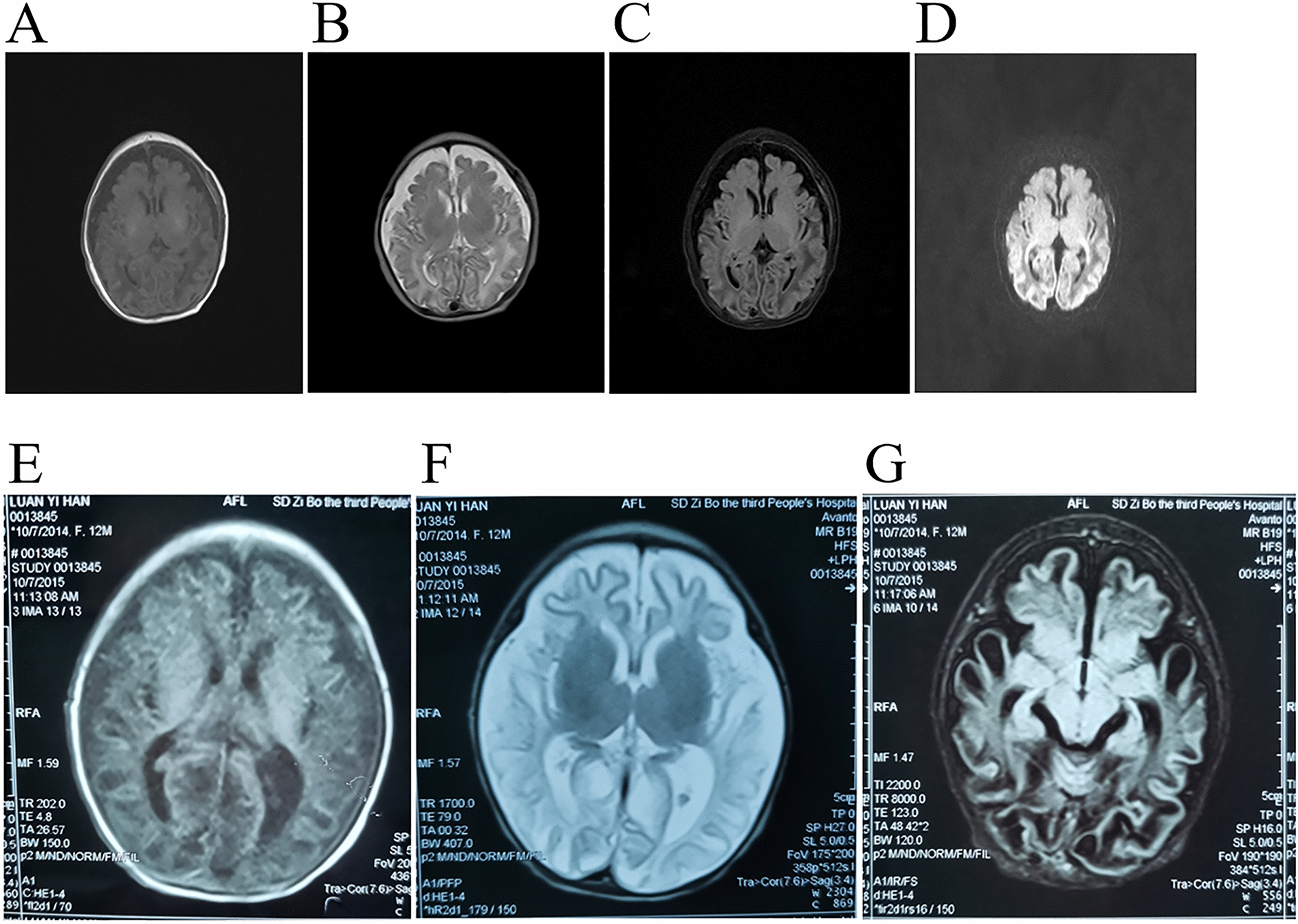
Brain MRI of the proband (**A**–**D)** and the elder sister (**E**–**G)**. **A** and **E** T1-weighted image (T1WI). **B** and **F** T2-weighted image (T2WI). **C** and **G** Fluid-attenuated inversion recovery (FLAIR) image. **D** Diffusion-weighted image (DWI)

Blood routine showed significantly elevated white blood cells. Biochemical results indicated that the levels of total protein, albumin and blood glucose were decreased, while the levels of total bilirubin, indirect bilirubin, lactate dehydrogenase, creatine kinase, and α-hydroxybutyrate dehydrogenase were increased. Blood gas analysis revealed that the lactate level was 3.2 mmol/L (normal range: 0.5–1.6 mmol/L). Chest X-ray showed increased bilateral lung texture in the lower lung fields. Cranial color ultrasound revealed abnormal right subependymal echo and subependymal bleeding was considered. Craniocerebral magnetic resonance imaging (MRI) indicated that large patchy of slightly long T1 and slightly long T2 signal shadows was seen in the brain parenchyma of bilateral cerebral hemispheres with unclear boundary. The cortex became thin. There were fissure-like long T1 and long T2 signal shadows in the bilateral external capsule with clear boundary. Patchy and striped DWI high signal shadows were observed in the white matter around the posterior horn of bilateral lateral ventricles. The bilateral ventricles were slightly enlarged, cerebral sulci, and fissure were widened and deepened, subarachnoid spaces were widened in frontal, parietal and temporal regions, and midline structure was in the middle (Fig. 2A-D).

The proband was diagnosed with neonatal pneumonia, neonatal encephalopathy, intracranial hemorrhage, neonatal hypoglycemia, and neonatal hyperbilirubinemia and was given symptomatic treatment and supportive care during hospitalization. He underwent WES for gene mutation screening to determine the clinical diagnosis. Four days after admission, his parents refused further treatment and asked to be discharged. Unfortunately, the proband died at 37 days of age.

His 32-day-old elder sister (Patient II: 1, Fig. 1), born at full term by cesarean section with a history of intrauterine asphyxia, was hospitalized for no weight gain for more than 1 month. She was given mixed feeding with feeding amount of 30 mL/2 h accompanied by nonprojectile vomiting. Physical examination showed pale skin, flat anterior fontanelle, normal auscultation of heart and lungs, soft abdomen and hypotonia of the limbs.

Laboratory examinations showed lower value of hemoglobin and increased levels of creatine kinase isoenzyme MB, γ-glutamyl transpeptidase, total bile acid, lactate dehydrogenase, α-hydroxybutyrate dehydrogenase, blood ammonia, aspartate aminotransferase, and alanine aminotransferase, supporting the diagnosis of liver dysfunction. Craniocerebral MRI showed unclear boundary between cortex and the medulla of the bilateral cerebral hemispheres; there was large patchy of long T1 and long T2 signal shadows in the bilateral frontal, parietal, and occipital lobes. In addition, the ventricles were enlarged, cerebral sulci and fissure were widened, and midline structure was in the middle (Fig. 2E–G). After severe hypoxic-ischemic encephalopathy and liver dysfunction were made at initial diagnosis, the patient was recommended to complete various examinations and be hospitalized. However, the parents refused to continue treatment, and the patient died at 34 days.

### Molecular genetic analyses

**Fig. 3:**
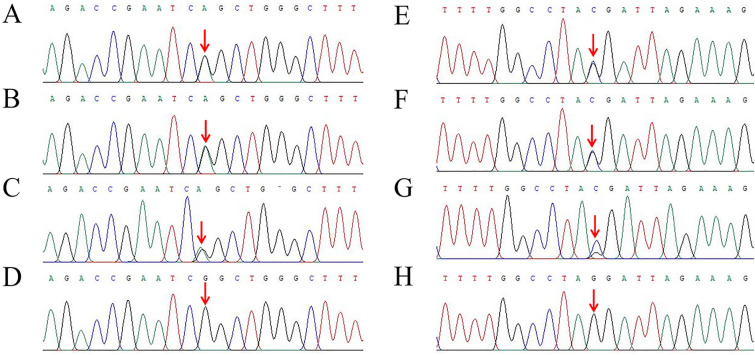
Partial DNA sequence chromatograms of *FARS2*. The red arrows represent the location of the variants c.925G>A and c.943G>C. **A** Heterozygous variant c.925G>A identified in this proband. **B** Heterozygous variant c.925G>A identified in the father. **C** Heterozygous variant c.925G>A identified in the elder sister. **D** Normal DNA sequence of the mother. **E** Heterozygous variant c.943G>C identified in this proband. **F** Heterozygous variant c.943G>C identified in the mother. **G** Heterozygous variant c.943G>C identified in the elder sister. **H** Normal DNA sequence of the father

In our study, one previously reported missense variant c.925G>A p.Gly309Ser [12] (Fig. 3A) and one novel missense variant c.943G>C p.Gly315Arg (Fig. 3E) in the *FARS2* gene (NM_006567.5) were detected in this proband by WES. Sanger sequencing revealed that the unaffected father (I: 1, Fig. 1) was a heterozygous carrier for p.Gly309Ser (Fig. 3B), while the asymptomatic mother (I: 2, Fig. 1) carried the heterozygous p.Gly315Arg variant (Fig. 3F). In addition, genetic analysis confirmed that his elder sister carried identical compound heterozygous variants of *FARS2* (Fig. 3C, G). Neither of the variants was found in 100 healthy unrelated controls of Chinese Han origin and has been reported in the gnomAD database. American College of Medical Genetics and Genomics (ACMG) guidelines [13] indicated that p.Gly309Ser was “likely pathogenic” and p.Gly315Arg was “uncertain” with evidence of PM2_Supporting + PM3_Strong + PP3_Moderate and PM2_Supporting + PP3_Moderate, respectively. In addition, p.Gly309Ser and p.Gly315Arg were classified as “Likely pathogenic” and “Uncertain significance,” respectively, using InterVar software based on ACMG guidelines [14]. In silico prediction revealed that the two *FARS2* variants were deleterious (Table 1). Based on these findings, the identified compound heterozygous *FARS2* variants were considered to be causative for disease phenotypes of this proband and his female sibling. They both were finally diagnosed with COXPD14 based on the clinical and laboratory findings as well as molecular genetic data.

**Table 1:** Pathogenicity analysis of the two *FARS2* variants c.925G>A and c.943G>C

Gene	Nucleotide change	Amino acid change	Status	Prediction tools					
				REVEL	PolyPhen-2	MutationTaster	SIFT	ClinPred	PROVEAN
*FARS2*	c.925G>A	p.G309S	Known	D (0.885)	Probably damaging (1)	Disease causing (1)	Damaging (0.006)	0.99848169	Deleterious (− 5.027)
*FARS2*	c.943G>C	p.G315R	Novel	D (0.921)	Probably damaging (1)	Disease causing (1)	Damaging (0)	0.99980956	Deleterious − (7.680)

### Sequence conservation analysis of mtPheRS protein

**Fig. 4:**
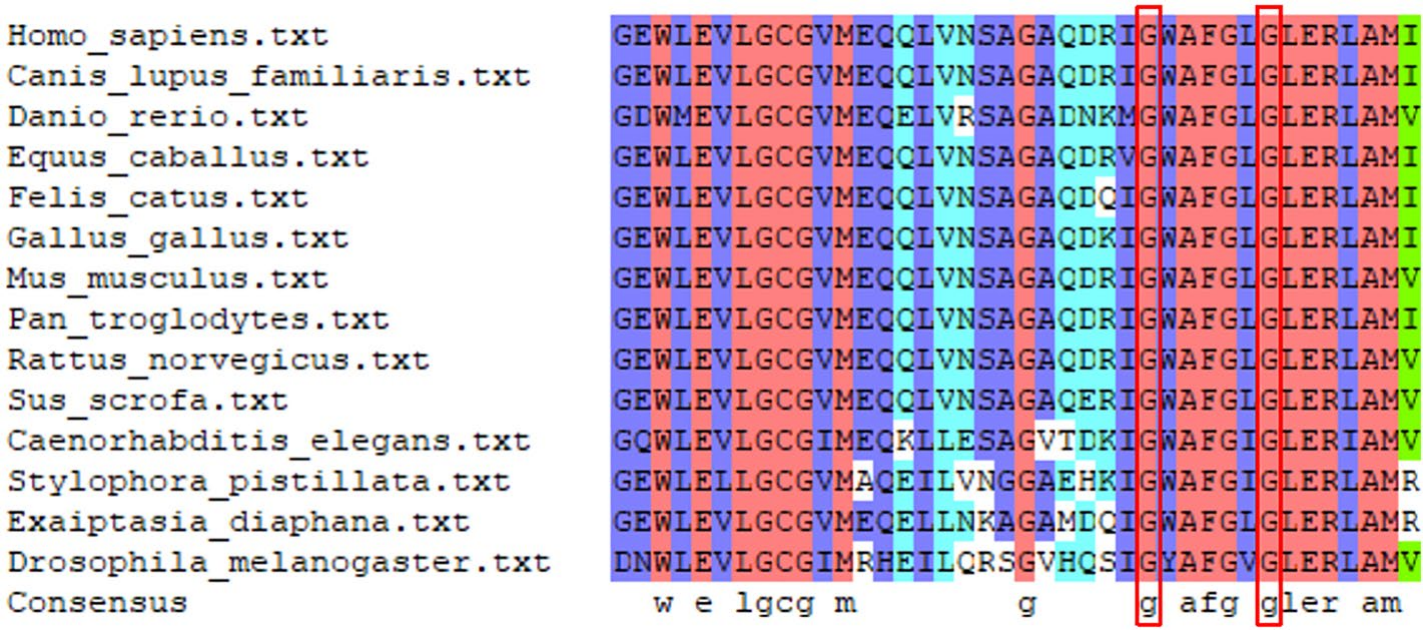
Protein sequence conservation analysis among different species. The red rectangles represent Gly309 and Gly315

The mtPheRS protein sequences of various species were obtained from the NCBI website. Sequence alignment of the mtPheRS protein sequences from these species was done by using the DNAMAN software, which revealed that Gly309 and Gly315 are both highly conserved residues (Fig. 4).

## Discussion

In the present study, we reported two siblings with autosomal recessive COXPD14 and identified two damaging compound heterozygous *FARS2* variants c.925G>A p.Gly309Ser and c.943G>C p.Gly315Arg by WES. The p.Gly309Ser variant has been reported previously, while p.Gly315Arg is a novel one. The patients who presented with hypoxic-ischemic encephalopathy, hypotonia of the limbs, and abnormal craniocerebral MRI findings were severely affected. In addition, a high lactate level was observed in this proband and the sister suffered from feeding difficulties, developmental delay, and abnormal liver function. However, both of them died more than 30 days after birth before they experienced seizures. Our study highlights that genetic testing is of great significance in the diagnosis of diseases with a wide variety of phenotypes, especially in the differential diagnosis of diseases, and can be served as a gold standard for diseases that cannot be made definitive diagnosis clinically.

COXPD14 is an unusual autosomal recessive disorder caused by defects in *FARS2* and is characterized by early-onset encephalopathy with or without epilepsy, developmental delay, high levels of lactate, and short or long lifetimes [15]. The first *FARS2* variant p.Tyr144Cys was reported by Shamseldin et al*.* in 2012, who described a 2-year-old female with clinical presentations of seizures, muscle weakness, developmental delay, and an increased lactate level [16]. Since then, a growing number of *FARS2* deleterious variants have been identified in three different disorders: COXPD14 with early-onset encephalopathy with or without epilepsy, COXPD14 with juvenile-onset epilepsy, and spastic paraplegia type 77 (SPG77, MIM: 617046) [9]. The disease type of the patients should be determined based on the main clinical findings combined with age at onset because patients can develop identical symptoms at different ages and phenotypes among these diseases overlap, such as increased lactate level, developmental delay, and seizures.

The clinical and genetic features of our patients and the previously reported cases with *FARS2* variants affected by early-onset encephalopathy, juvenile-onset epilepsy, and spastic paraplegia were summarized in Tables 2, 3, and 4, respectively, after literature review. Approximately 44 subjects with disease-causing variants in *FARS2* have been reported, including 27 cases of early-onset encephalopathy with or without epilepsy, 3 cases of juvenile-onset epilepsy, and 14 cases of spastic paraplegia.

**Table 2:** The clinical and genetic features of our patients and the previously reported cases with *FARS2* mutations affected by early-onset encephalopathy

References	Subject	Ethnicity	Consanguinity	Gender	Seizures and age of onset	Brain MRI	Other clinical phenotypes	Death of age	*FARS2* variants
This study	1	Chinese	No	M	No	Long T1 and long T2 signal shadows in the brain parenchyma of bilateral cerebral hemispheres, cortical thinning, long T1 and long T2 signal shadows in the bilateral external capsule, DWI high signal shadows in the white matter around the posterior horn of bilateral lateral ventricles, enlargement of the ventricles, widened and deepened cerebral sulci and fissure, widened subarachnoid spaces in frontal, parietal, and temporal regions	Hypoxic-ischemic encephalopathy, hypotonia of the limbs, a high lactate level	37 days	p.G309S/p.G315R (het)
	2	Chinese	No	F	No	Unclear corticomedullary demarcation of the bilateral cerebral hemispheres, long T1 and long T2 signal shadows in the bilateral frontal, parietal and occipital lobes, enlargement of the ventricles, widened cerebral sulci and fissure	Hypoxic-ischemic encephalopathy, hypotonia of the limbs, feeding difficulties, developmental delay and abnormal liver function	34 days	p.G309S/p.G315R (het)
Shamseldin et al. [16]	3	Saudi	Yes	F	Seizures, myoclonus, NA	Similar to MRI findings of Leigh syndrome	Muscle weakness, developmental delay, lactic acidosis	22 months	p.Y144C (hom)
Elo et al. [18]	4	Finnish	No	F	Myoclonic jerks, 2 days	Severe central and cortical atrophy with slight bilateral signal increase in the putamina	Elevated lactate, microcephaly, narrowed, and atrophic gyri	8 months	p.I329T/p.D391V (het)
	5	Finnish	No	F	Seizures, 4 days	NA	Elevated lactate	21 months	p.I329T/p.D391V (het)
Almalki et al*. *[10]	6	White British	No	M	Infantile spasms, 6 months	Symmetrical subcortical white matter lesions with thinning of the anterior and genu of the corpus callosum	Developmental delay, small, round, anteriorly rotated ears, and a broad nasal root	Alive at 30 months of age	p.D325Y/an 88 kb microdeletion (het)
Cho et al*. *[12]	7	Korean	No	M	Generalized tonic–clonic seizures, 3 months	A diffusely atrophic brain at 3 months; Progression of atrophic changes and myelination delay at 6 months	Hypotonia, delayed motor development, spastic four extremities, and increased deep tendon reflexes	Alive at 3 years of age	p.G309S (hom)
	8	Korean	No	F	Myoclonic movement starting from the right hand and being generalized to the entire body, 4 months	A thin corpus callosum and generalized brain atrophy	NA	Alive at 17 months of age	p.G309S (hom)
	9	Korean	No	M	Infantile spasms, 4 months	Mild brain atrophy	Abnormal liver function, an elevated lactate level	8 months	p.G309S (hom)
	10	Korean	No	F	Generalized tonic–clonic seizures, 3 months	Mild brain atrophy	Abnormal liver function, an elevated lactate level	4 months	p.G309S (hom)
Raviglione et al*. *[7]	11	Romanian	No	M	Infantile spasms, 3 months	Microcephaly, enlargement of frontal subarachnoid spaces, and lateral ventricles due to a reduction in volume of the cerebral white matter, slight hyperintensity of hemispheric white matter on T2-weighted images, thin corpus callosum, thinning of the cortical rim	Psychomotor delay, microcephaly, widely spaced eyes, large ears, bilateral divergent strabismus with visual impairment, and bilateral horizontal nystagmus, axial hypotonia and mild distal hypertonia	Alive at 3 years of age	p.R386G/a 134 kb microdeletion (het)
Almannai et al*. *[6]	12	Arab	Yes	F	Seizures, NA	Brain atrophy, thin corpus callosum	Developmental delay, microcephaly, liver disease, elevated lactate	23 months	p.Y144C (hom)
	13	Arab	Yes	F	Seizures, NA	Brain atrophy	Developmental delay, microcephaly, liver disease, elevated lactate	3 months	p.Y144C (hom)
	14	Arab	Yes	M	Seizures, 1 month	Brain atrophy, thin corpus callosum	Developmental delay, microcephaly, liver disease, elevated lactate	Alive at 2 years of age	p.Y144C (hom)
	15	Arab	NA	F	Seizures, 2 months	NA	Developmental delay, elevated lactate	NA	p.Y144C (hom)
	16	Arab	No	F	Seizures, 1 month	Brain atrophy, thin corpus callosum	Developmental delay, microcephaly, liver disease, elevated lactate	Alive at 1 year of age	p.Y144C (hom)
	17	Arab	Yes	M	Seizures, 1 month	Thin corpus callosum	Developmental delay, liver disease, elevated lactate	3 months	p.Y144C (hom)
	18	Arab	No	F	Seizures, 1 month	Brain atrophy, thin corpus callosum	Developmental delay, microcephaly, liver disease, elevated lactate	Alive at 13 months of age	p.Y144C (hom)
	19	Arab	Yes	F	Seizures, 5 months	Brain atrophy, thin corpus callosum	Developmental delay, microcephaly, liver disease, elevated lactate	2 years	p.Y144C (hom)
	20	Arab	Yes	F	Seizures, 1 month	Brain atrophy, thin corpus callosum	Developmental delay, microcephaly, liver disease, elevated lactate	Alive at 4.5 months of age	p.Y144C (hom)
	21	Arab	Yes	F	Seizures, 20 days	Brain atrophy, thin corpus callosum	Developmental delay, microcephaly, liver disease, elevated lactate	4 months	p.Y144C (hom)
	22	Arab	Yes	F	Seizures, 25 days	Brain atrophy, thin corpus callosum	Developmental delay, microcephaly, liver disease, elevated lactate	3.5 months	p.Y144C (hom)
	23	Arab	Yes	F	No	NA	Liver disease, elevated lactate	2 days	p.V177D/p.Y144C (het)
	24	Spanish	NA	F	NA	NA	Elevated lactate	NA	p.G309S/p.R153G (het)
Barcia et al*. *[15]	25	French and Chinese	No	F	No	Mild ventriculomegaly	Axial hypotonia, developmental delay, and spastic tetraparesis	Alive at 8 years of age	p.R419H/p.S426^*^
	26	French	No	F	Myoclonic focal and generalized seizures, 19 months	Marked ventriculomegaly, enlargement of the subarachnoid spaces due to white matter loss, especially in the Sylvian fissures, abnormal T2 hyperintensities in the lentiform nuclei and dorsal brainstem, cerebellar atrophy	Global hypotonia, psychomotor delay, mild scoliosis, spastic tetraparesis, and severe muscular atrophy predominating on inferior limbs	Alive at 16 years of age	p.R330H/p.L371F (het)
	27	French	No	M	Myoclonic generalized and focal seizures, 1 year	Moderate ventriculomegaly and enlargement of the subarachnoid spaces; Dentate nuclei, brainstem and pallidal T2 hyperintensity	Severe psychomotor delay, global hypotonia and lumbar mild scoliosis	Alive at 5 years of age	p. R330H/p.L371F (het)

*M* male, *F* female, *MRI* magnetic resonance imaging, *het* heterozygous, *hom* homozygous

**Table 3:** The clinical and genetic features of the previously reported cases with FARS2 mutations affected by juvenile-onset epilepsy

References	Subject	Ethnicity	Consanguinity	Gender	Seizures and age of onset	Brain MRI	Other clinical phenotypes	Death of age	*FARS2* variants
Walker et al*. *[17]	1	NA	No	F	A prolonged generalized tonic–clonic convulsion, 8 years	Extensive areas of abnormal T2 hyperintensity in the frontal lobes (right greater than left), anterior cingulate gyri, left superior frontal gyrus, bilateral temporal lobes, and left cerebellar cortex	Motor and speech delays	15 years	p.P85A/p.H135D (het)
Hotait et al. [11]	2	NA	No	F	Brief focal aware clonic seizures semiologically characterized by twitching of the left side of the face, 16 years	Restricted diffusion in the cortical-subcortical areas of the right frontal lobe, right insula, right thalamus and to lesser extent in the right temporal, both parietal lobes and left frontal lobe	Paresis of left upper extremity	Alive at 17 years of age	p.V197M/exon 2 microdeletion (het)
Chen et al*. *[9]	3	NA	No	M	Generalized tonic–clonic convulsions, 12 years	Increased wandering lesions involving bilateral frontal, temporal, and parietal lobes, occipital cortex and subcortical	Increased serum lactic acid, pes cavus, mild muscular atrophy and compensatory hypertrophy	20 years	p.V197M/p.F402S (het)

*M* male, *F* female, *MRI* magnetic resonance imaging, *het* heterozygous

**Table 4:** The clinical and genetic features of the previously reported cases with *FARS2* mutations affected by spastic paraplegia

References	Subject	Ethnicity	Consanguinity	Gender	Seizures and age of onset	Brain MRI	Other clinical phenotypes	Death of age	*FARS2* variants
Yang et al*. *[19]	1	Chinese	Yes	F	No	Normal	Progressive lower limb spasticity, pyramidal weakness with hyperreflexia, extensor plantar responses, and scissors gait	Alive at 41 years of age	p.D142Y (hom)
	2	Chinese	Yes	M	No	Normal	Progressive lower limb spasticity, pyramidal weakness with hyperreflexia, extensor plantar responses, and scissors gait	Alive at 30 years of age	p.D142Y (hom)
	3	Chinese	Yes	F	No	Normal	Progressive lower limb spasticity, pyramidal weakness with hyperreflexia, extensor plantar responses, and scissors gait	Alive at 26 years of age	p.D142Y (hom)
	4	Chinese	Yes	F	No	Normal	Progressive lower limb spasticity, pyramidal weakness with hyperreflexia, extensor plantar responses, and scissors gait	Alive at 23 years of age	p.D142Y (hom)
Vantroys et al. [20]	5	NA	No	M	Convulsive seizures, 19 months	Slight cortical atrophy at 20 months; Bilateral, round, focal T2-hyperintense lesions in the anterior part of the mesencephalon at 17 years	Increased lactate, developmental delay, spastic paraplegia, neurogenic bladder, and sphincter dyssynergia	Alive at 19 years of age	p.A154V/p.P361L (het)
	6	NA	NA	F	No	Symmetrical T2 hyperintensities of the posterior tegmentum at 17 months; More extensive T2 hyperintense lesions at the tegmentum and periaqueductal gray matter at 6 years; Near resolution of the tegmental lesions but new T2 hyperintense lesions bilaterally in the anterior inferior thalamus and signs of cerebellar atrophy at 15 years	Delayed motor development, spastic paraplegia	Alive at 15 years of age	p.V174del/p.P361L (het)
Vernon et al*. *[8]	7	NA	NA	F	Seizure, 2 months	Normal	Globally delayed development, mild facial dysmorphism, an elevated lactate level, truncal hypotonia with brisk extremity reflexes throughout, and an intermittent intention tremor	Alive at 5 years of age	p.R419C/a 116 kb microdeletion (het)
	8	NA	NA	M	Seizures, only within 6 weeks after birth	Two small foci of T2/FLAIR hyperintensity involving the periventricular white matter and deep white matter of the right posterior frontal lobe	Delayed development, cerebral palsy, metabolic acidosis, truncal hypotonia, dysarthric speech, and a mild intention tremor	Alive at 13 years of age	p.R419C/a 116 kb microdeletion (het)
Almannai et al. [6]	9	North American	No	F	No	Brain atrophy	Developmental delay, spastic paraplegia	Alive at 20 years of age	p.H159P/p.R419C (het)
	10	North American	No	F	No	NA	Developmental delay, spastic paraplegia	Alive at 17 years of age	p.H159P/p.R419C (het)
Sahai et al*. *[21]	11	Northern European and Ashkenazi Jewish	No	M	No	Abnormal signal hyperintensities in the bilateral dentate nuclei	Spasticity in lower extremities	Alive at 9 years of age	p.Q216X/p.P136H (het)
Meszarosova et al*. *[22]	12	Czech Roma	No	M	No	Normal	Gait impairment, progressive limb spasticity, hyperreflexia, pes cavus	Alive at 22 years of age	p.P361L/exons 1–2 microdeletion (het)
Forman et al. [23]	13	Irish	No	M	No	Normal	Delayed walking, tremor in the upper limbs, dysphonia; Spasticity, weakness, brisk deep tendon reflexes, extensor plantar responses, and clonus in the lower limbs	Alive at 13 years of age	p.G141E/an 75 kb microdeletion (het)
	14	Irish	No	F	No	Normal	Delayed walking, tremor in the upper limbs; Spasticity, weakness, brisk deep tendon reflexes, extensor plantar responses, and clonus in the lower limbs	Alive at 7 years of age	p.G141E/an 75 kb microdeletion (het)

*M* male, *F* female, *MRI* magnetic resonance imaging, *het* heterozygous, *hom* homozygous

In our patients and the reported cases in the literature with early-onset encephalopathy, although the majority of patients died within 2 years of age, we found that some could survive beyond the age of 2, with the oldest surviving at age 16. Barcia et al*.* have described three early-onset patients with or without epileptic seizures, all of whom had longer lifespans and they were still alive at the time of the study, which highlighted that not all patients with early-onset form experience seizures or have poor outcomes [15]. To date, COXPD14 with juvenile-onset epilepsy was found in only three individuals. The first case exhibited developmental delay and died of likely pneumonia and urinary tract infections at the age of 15 years [17], whereas another patient reported by Chen et al*.* in 2109 had normal development but failed to survive due to pulmonary infection at age 20 years [9]. For patients suffered from spastic paraplegia, they showed the lowest disease severity and had a good prognosis in comparison with the other two disorders, with many being able to survive into adulthood. Interestingly, all cases with spastic paraplegia in the literature were alive at the time of report.

A total of 32 different *FARS2* variants were discovered so far, including 23 missense variants, 1 three-base-pair deletion, 1 eight-base-pair duplication, 1 nonsense variant, and 6 microdeletions. The microdeletions were associated with three *FARS2*-related disorders and all were combined with missense variants to form compound heterozygous states. Similarly, patients with deletion, duplication, and nonsense variants also had a heterozygous missense variant. Both of the mutation sites identified in this study were evolutionarily conserved and located in the catalytic domain, which resulted in severe phenotypes of the two siblings. Nevertheless, the causal correlation between the protein domains affected by the mutation sites and disease phenotypes or severity remains unclear. Patients suffering from COXPD14 with early-onset encephalopathy had variants in the catalytic domain, linker region, and anticodon-binding domain, while COXPD14 with juvenile-onset epilepsy and SPG77 were associated with variants in the catalytic and anticodon-binding domains.

Of all the variants, only three have been presented in homozygous states. Both homozygous p.Gly309Ser and p.Tyr144Cys could give rise to early-onset epileptic encephalopathy, while patients with homozygous p.Asp142Tyr manifested spastic paraplegia. The *FARS2* variant p.Gly309Ser was revealed as a Korean founder pathogenic variant and p.Tyr144Cys was a founder variant in Arabs despite the fact that it was initially identified in a Saudi female patient. All Arab patients presented by Almannai et al*.* carried this variant, 11 out of the 12 subjects were homozygotes and only one patient were compound heterozygous for p.Tyr144Cys and p.Val177Asp [6]. Different variants in the same locus may be responsible for distinct phenotypes. For example, the p.Arg419His variant is linked to early-onset encephalopathy without seizures [15], whereas p.Arg419Cys was only reported in cases with spastic paraplegia [6, 8]. The same variant could be compound heterozygous with other different variants. The variant p.Val197Met could be in combination with 1 exon 2 microdeletion or one p.Phe402Ser variant, respectively. In addition, for our patients and the four Korean patients described by Cho et al.[12], because they all harbored identical variant p.Gly309Ser, both presented with similar clinical features, such as abnormal brain MRI, elevated lactate level, hypotonia, developmental delay, and liver dysfunction. However, disease severity between the two groups could not be compared. The two siblings in our study passed away soon after birth due to severe clinical manifestations and treatment abandonment, and therefore, we failed to follow the disease process. The other variant in our patients and the four Korean patients suggested that disease severity may vary, but this needed to be further evaluated and validated.

In summary, our study revealed the genetic basis and clinical features of two Chinese siblings with COXPD14 and expanded the mutation spectrum of *FARS2*. The two compound heterozygous variants in *FARS2* are associated with the phenotypic characteristics of the patients. However, further research is essential to explore the pathogenesis of COXPD14 caused by dysfunction of mtPheRS protein resulting from *FARS2* variants.

## Data Availability

The data analyzed during this study are available from the corresponding authors upon reasonable request.
